# Effects of disease severity distribution on the performance of quantitative diagnostic methods and proposal of a novel ‘V-plot’ methodology to display accuracy values

**DOI:** 10.1136/openhrt-2017-000663

**Published:** 2018-01-20

**Authors:** Ricardo Petraco, Hakim-Moulay Dehbi, James P Howard, Matthew J Shun-Shin, Sayan Sen, Sukhjinder S Nijjer, Jamil Mayet, Justin E Davies, Darrel P Francis

**Affiliations:** International Centre for Circulatory Health, National Heart and Lung Institute, Imperial College London and Imperial College Healthcare NHS Trust, London, UK

**Keywords:** diagnostic accuracy, diagnostic tests, study sample

## Abstract

**Background:**

Diagnostic accuracy is widely accepted by researchers and clinicians as an optimal expression of a test’s performance. The aim of this study was to evaluate the effects of disease severity distribution on values of diagnostic accuracy as well as propose a sample-independent methodology to calculate and display accuracy of diagnostic tests.

**Methods and findings:**

We evaluated the diagnostic relationship between two hypothetical methods to measure serum cholesterol (Chol_rapid_ and Chol_gold_) by generating samples with statistical software and (1) keeping the numerical relationship between methods unchanged and (2) changing the distribution of cholesterol values. Metrics of categorical agreement were calculated (accuracy, sensitivity and specificity). Finally, a novel methodology to display and calculate accuracy values was presented (the V-plot of accuracies).

**Conclusion:**

No single value of diagnostic accuracy can be used to describe the relationship between tests, as accuracy is a metric heavily affected by the underlying sample distribution. Our novel proposed methodology, the V-plot of accuracies, can be used as a sample-independent measure of a test performance against a reference gold standard.

**Key questions**

**What is already known about this subject?**

Diagnostic methods are often chosen by clinicians based on their published diagnostic accuracy against a reference gold standard. The diagnostic accuracy of a method is known to be affected by the underlying disease prevalence.

**What does this study add?**

Using a practical example, we demonstrate how the pattern of disease severity distribution (intermediate vs extreme values) can affect the diagnostic accuracy of a method beyond disease prevalence. We propose the use of a novel V-plot of accuracies to display diagnostic accuracy values, which is a sample-independent, universal measure of a method’s categorical agreement with a reference standard.

**How might this impact on clinical practice?**

Using the V-plot of accuracies, researchers can describe the categorical agreement between two methods of measurement, and physicians may immediately appreciate the upper and lower limits of a test’s accuracy to decide which diagnostic method to choose for their patients.

## Introduction

Almost all clinically useful biological measurements are quantifiable continuous variables, such as arterial blood pressure, plasma glucose and serum cholesterol. For clinical convenience, however, many are interpreted qualitatively by a dichotomous classification into *normal* versus *abnormal,* based on a fixed cut-off. For instance, although serum levels of cholesterol can be quantified and displayed across a wide spectrum of values, patients are often labelled as having *hypercholesterolaemia* versus *normal cholesterol* based on an accepted cut-off value. Dichotomising results into positive versus negative is common in clinical practice because of the perceived pressure of information overload and with the reason given that often clinical decisions are themselves dichotomous (treat vs not treat, start or not a statin).

**Figure 1:**
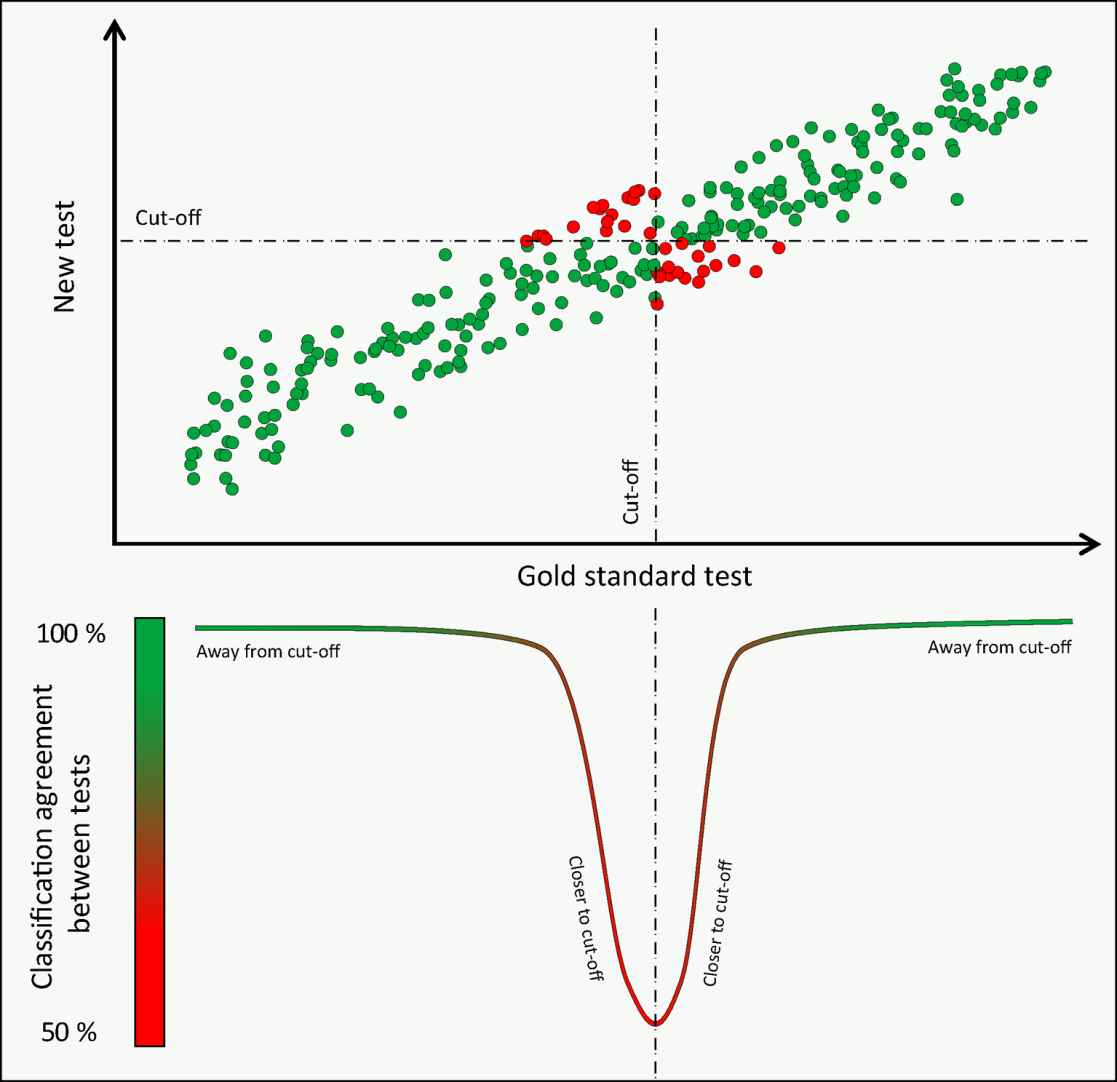
Disease severity and classification agreement between methods: schematic representation of the principle that classification agreement between two methods of measurement (or diagnostic accuracy if one is seen as a reference gold standard) varies across the range of disease severity. At the extremes of disease and health agreement is 100%. Close to the classification cut-off, around the intermediate range of disease severity, agreement falls, reaching a nadir close to 50%.

Qualitatively, the performance of a diagnostic test against a reference gold standard can be quantified by its *diagnostic accuracy* and the directly related *sensitivity*, *specificity*, *predictive values*, *likelihood ratios* and *area under receiver operator characteristic (ROC) curve.* 1 Among all such available measures, physicians often choose diagnostic methods based on their published accuracy, a demonstration that statistical concepts can directly influence patient care and healthcare policies.^2^ However, relying on a universal value of diagnostic accuracy as an idealised measure of a test’s performance is an approach with known limitations. First, accuracy values are known to depend on the prevalence of disease in the underlying sample, an extensively explored phenomenon.^3 4^ Second, beyond disease prevalence, *how* disease severity is distributed within a study sample independently affects a test’s accuracy, a phenomenon rarely discussed^4 5^ and schematically explained in figure 1. For any given quantitative diagnostic method and its reference comparison, diagnostic accuracy can have any value from approximately 50% up to 100%; this value will depend on the numerical agreement between the methods (how good a test is) and also on whether the sample studied is formed by intermediate (close to cut-off) or extremes (away from cut-off) forms of disease.

In the present study, we aim to explore the specific effect of disease severity distribution on values of diagnostic accuracy and related statistical measures. As a solution, we propose the use of the accuracy V-plot, a novel, sample-independent method to calculate and present accuracy values.

## Methods

### Hypothetical studies on a new method to measure serum cholesterol

This study used a hypothetical comparison between a new method to measure serum cholesterol (Chol_rapid_) and an established gold standard (Chol_gold_). The relationship between the two methods was compared in two different samples, artificially generated using statistical software (Mathworks, Natick, Massachusetts, USA):

1. *Validation study sample*: generated from 238 random values of cholesterol (Chol_gold_), not normally distributed, ranging from 2.9 mmol/L to 8.9 mmol/L, with a mean of 5.9 mmol/L.
2. *Primary care, clinical study sample*: generated from a narrower spread of 987 random, normally distributed values of cholesterol (Chol_gold_), ranging from 3.6 mmol/L to 7.6 mmol/L, with a mean of 5.8 mmol/L and SD of 0.58 mmol/L.

In both samples 1 and 2, Chol_rapid_ values were randomly generated keeping the mean difference with Chol_gold_ close to 0 (0.05 mmol/L for sample 1 and 0.02 mmol/L for sample 2) and using a fixed SD of the difference (SDD) between methods (SDD sample 1=0.36 mmol/L and SDD sample 2=0.35 mmol/L).

The relationship between the two methods (Chol_rapid_ and Chol_gold_) was then compared in each sample using the following parameters:

1. Numerical relationship was evaluated using scatter plots and Bland-Altman plots.
2. Categorical relationship: diagnostic accuracy (or classification agreement between methods, defined as the total number of correctly classified data points divided by the total number of data points), sensitivity, specificity, positive and negative predictive values, positive and negative likelihood ratios and area under ROC curves. For the qualitative analysis, an arbitrary value of 5.7 mmol/L for Chol_gold_ and Chol_rapid_ values was defined as normal.

### The V-plot of accuracies

We introduce a novel methodology to calculate and present classification agreement (accuracy) between methods, whereby the accuracy of Chol_rapid_ is calculated and displayed across multiple quantiles of disease severity (from 2 to 10 mmol/L in 1 mmol/L bands). Finally, we proposed a method to estimate the accuracy of Chol_rapid_ in independent samples in which the frequency distribution of Chol_gold_ is known (a detailed stepwise approach to V-plot derivation is presented in figures 6 and 7).

## Results

The characteristics of the samples generated as well as the relationship between Chol_rapid_ and Chol_gold_ in each sample are presented as a series of hypothetical studies to facilitate the interpretation of our results into clinical practice.

### A new diagnostic method for the screening of hypercholesterolaemia

Imagine investigators developed a new method to measure serum cholesterol that uses an infrared scan of the finger and yields an immediate value. The expectation was that this new test (Chol_rapid_) could be used in the primary care to screen for hypercholesterolaemia without the need for a needle or formal laboratory test and would enable identification of patients at high risk of cardiovascular events and lead to early initiation of therapy.

An initial large validation study was required before its implementation in clinical practice, so Chol_rapid_ had to be tested against the gold standard method of measuring cholesterol in the biochemistry laboratory (Chol_gold_). The validation study tested Chol_rapid_ performance across a wide range of cholesterol values. Therefore, 238 patients were recruited from multiple clinical settings: healthy young volunteers with no history of cardiac disease, patients with multiple risk factors from a cardiovascular clinic and patients from a specialised hyperlipidaemia outpatient service. For the purpose of diagnostic classification, a cholesterol result of 5.7 mmol/Lor above was considered hypercholesterolaemia.

**Figure 2:**
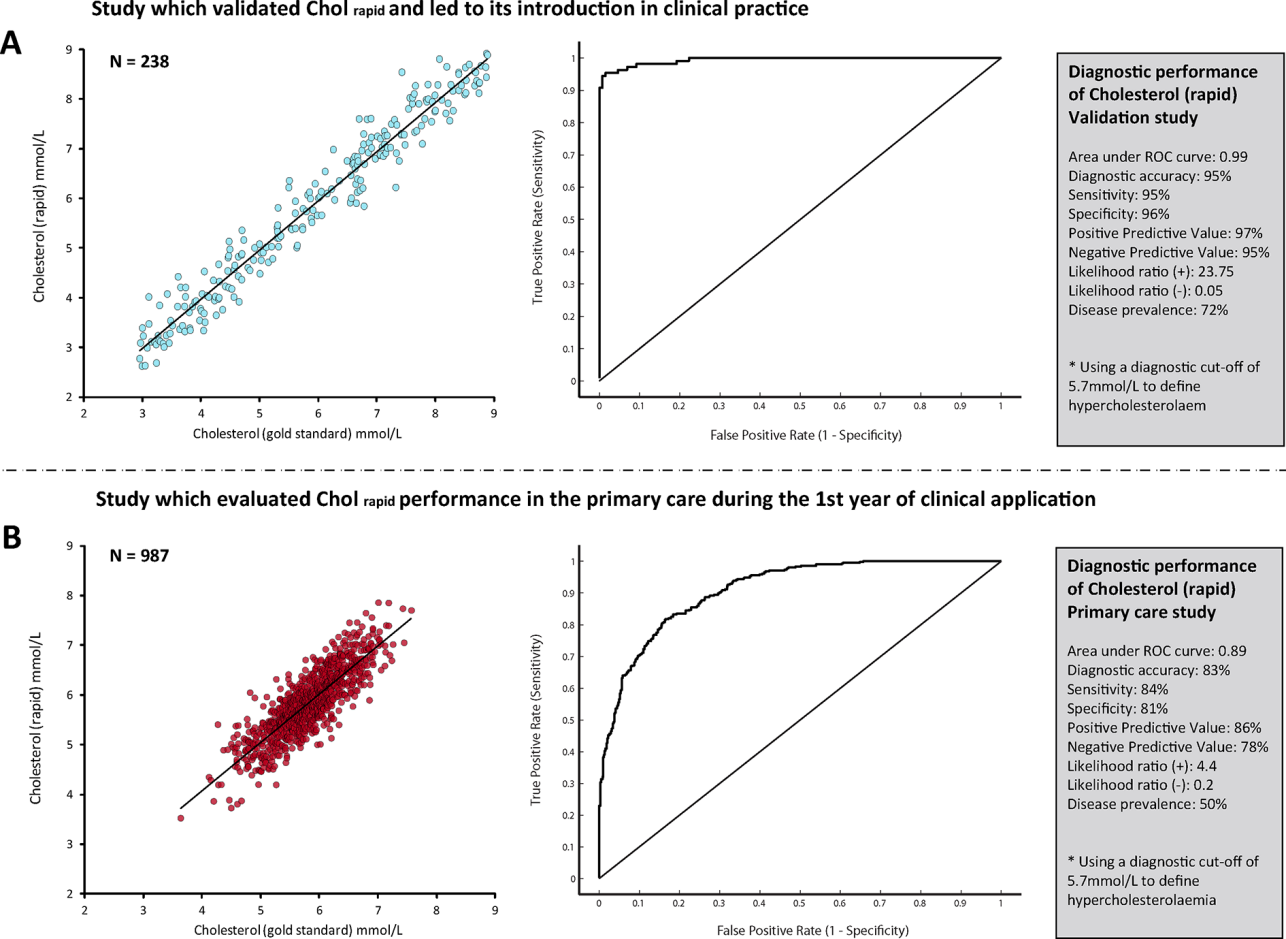
Diagnostic performance of the new cholesterol test. The performance of the new cholesterol test (Chol_rapid_) changed significantly between the two studies. The overall accuracy of Chol_rapid_ to diagnose hypercholesterolaemia fell in the primary care retrospective cohort (B), when compared with the initial validation study (A). Values of area under ROC curve, sensitivity, specificity and predictive values were also largely different. ROC, receiver operator characteristic.

The results of this initial study confirmed early expectations, with Chol_rapid_ showing an accuracy of 95% to diagnose hypercholesterolaemia, with a sensitivity of 95% and area under the ROC curve of 0.99 and positive and negative likelihood ratios of 23.75 and 0.05, respectively. Figure 2A summarises Chol_rapid_ diagnostic performance.

As a result, Chol_rapid_ was approved to be implemented in a large primary care unit for a period of trial. For 1 year, patients from the community with at least one risk factor for cardiovascular disease started having their cholesterol measured with Chol_rapid_. During this initial clinical evaluation, however, blood samples were still sent for standard laboratory analysis (Chol_gold_), for a period of real-world comparison.

At the end of the first year of its utilisation, investigators re-evaluated Chol_rapid_ diagnostic performance, comparing it against the same gold standard measurement Chol_gold_. The results of this second retrospective analysis were very disappointing. Chol_rapid_ diagnostic accuracy to identify patients with hypercholesterolaemia fell to 83%, with a significant drop in sensitivity (84%), area under ROC curve (0.89) as well as a significant change in positive and negative likelihood ratios (4.4 and 0.2, respectively) (figure 2B). As a result, a primary care safety committee decided to temporarily withhold Chol_rapid_ utilisation until a comprehensive assessment of its reliability was carried out.

The health authority looked into the reasons for such discrepancy between the final validation study and its first year of implementation but found nothing obvious: the technique applied was exactly the same, with comparisons made against Chol_gold_ tested in the same biochemistry laboratory.

**Figure 3:**
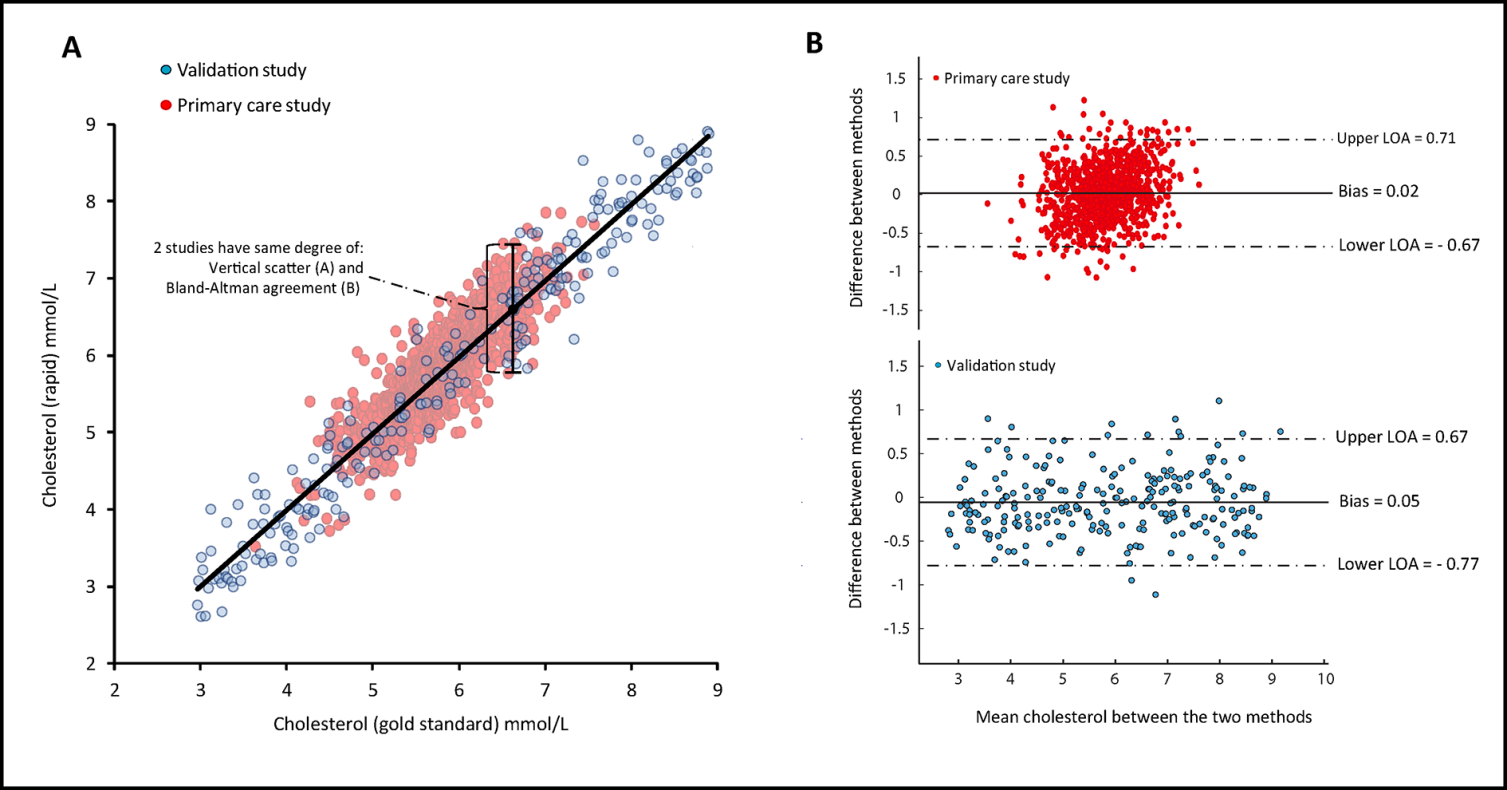
Numerical agreement between Chol_rapid_ and Cho_gold_ is equal between studies. Despite different magnitudes of classification agreement (diagnostic accuracy) between Chol_rapid_ and Chol_gold_ in the two studies, the raw measurement disagreement between the two methods remained unchanged. This can be appreciated from measures of the vertical scatter such as the SE of the estimate (plot A) and from Bland-Altman plots (B). It can be inferred that the observed drop in Chol_rapid_ performance in the primary care study cannot be explained by a change in its true measurement performance. LOA, limits of agreement.

The fundamental relationship between Chol_rapid_ and Chol_gold_ remained unaltered in the two studies, as shown by the degree of vertical dispersion of values (raw measurement disagreement) in both scatter plots (figure 3A). The stable relationship between the two methods can also be demonstrated in the form of Bland-Altman plots (figure 3B), which reveals that the 95% limits of agreement were almost identical in the two studies.^6^

**Figure 4:**
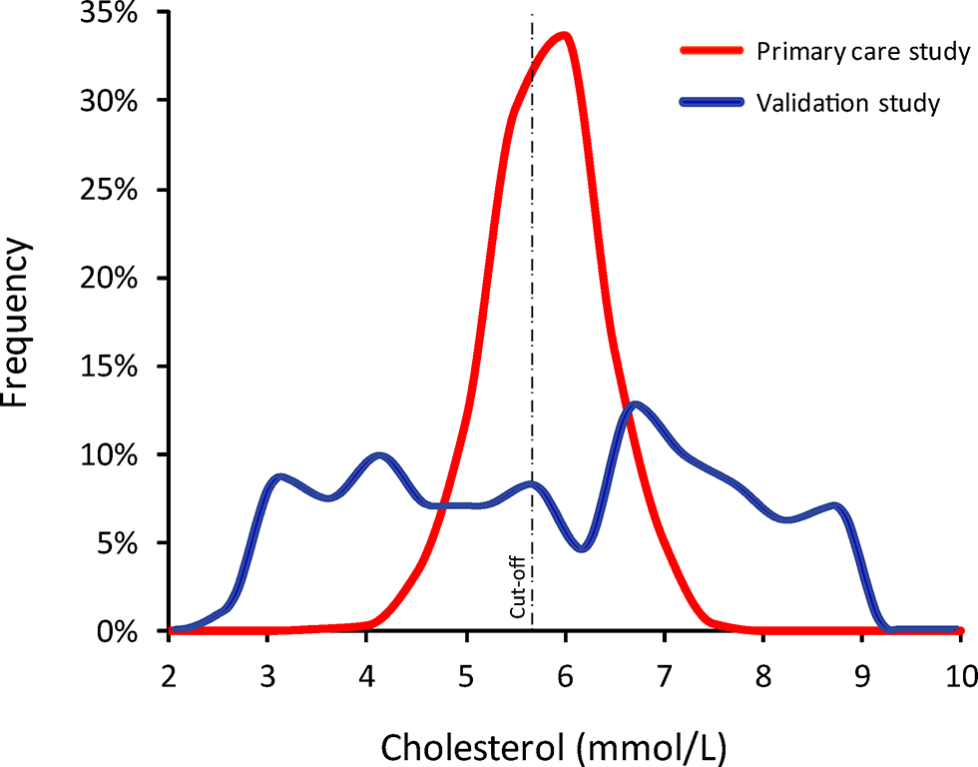
Histograms of cholesterol values from both studies. While the validation study included patients with a wide range of cholesterol values, the primary care cohort was formed predominantly of patients with intermediate values of cholesterol. This difference was responsible for the significant drop in Chol_rapid_ accuracy reported in the primary care study.

Therefore, the significant reduction in Chol_rapid_ diagnostic performance between studies (accuracy, ROC curve, sensitivity and so on) can be entirely explained by how differently cholesterol values were distributed in the two samples (figure 4). The specific explanation is that the studies differed significantly in what proportion of patients had cholesterol values close to the diagnostic cut-off of 5.7 mmol/L; while the initial validation study included patients with a wide range of cholesterol values (and so a large proportion of them far away from the cutpoint), the primary care study was mainly formed by patients with intermediate values of cholesterol, straddling the cut-off value, that is, the region where most disagreements occur. Differences in the distribution of cholesterol values, rather than in the actual measurement performance of Chol_rapid_, were responsible for the different accuracy values (figure 1).

### The V-plot: a per-range display of accuracy values

**Figure 5:**
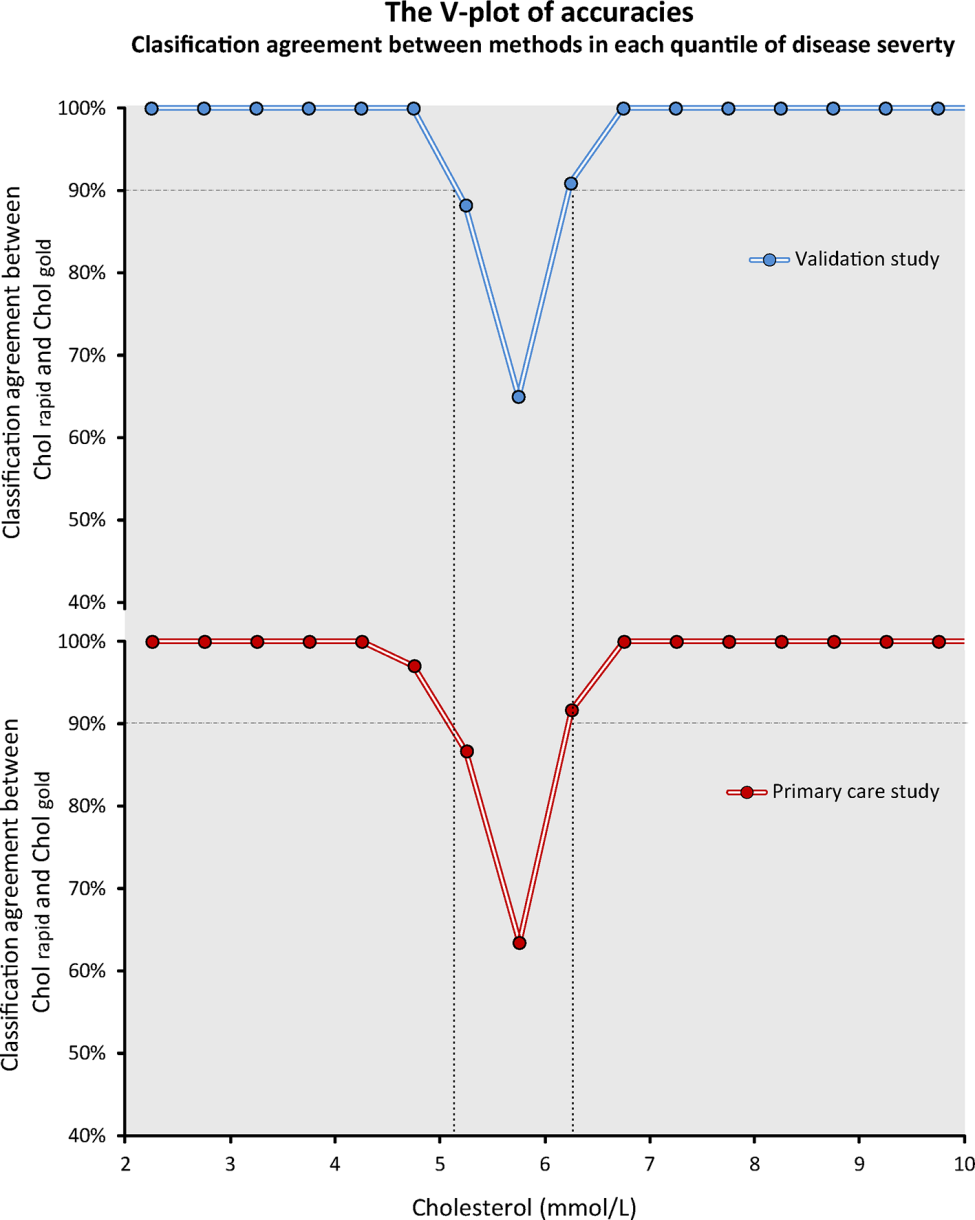
The V-plot of accuracies of Chol_rapid_. The V-plot permits a visual demonstration that the classification agreement between Chol_rapid_ and Chol_gold_ is equal in the two studies in each quantile of disease severity. The *overall* classification agreement (diagnostic accuracy of Chol_rapid_) could change between studies, depending on the proportion of patients in each quantile. The V-plot consistently identifies the range of cholesterol values within which the agreement between tests is lower than 90% (dashed lines).

To circumvent the sample dependency of overall accuracy values, instead of simply calculating an *overall* value of diagnostic accuracy for the whole study population, we propose to calculate the classification agreement between methods in *each part of the spectrum* of disease severity. This results in several *per-quantile* values of accuracies, which can be displayed across the entire range of disease severity to generate a V-shaped plot, which gives name to the method (figure 5). The V-plot has this shape because the accuracy of tests is universally high at the extremes of disease severity (near 100%) but close to the classification cut-off agreement plunges to around 50%. The width of the mouth of the V can be used as a general measure of a test’s performance: the wider the V, the poorer the test ability to match a reference modality.

**Figure 6:**
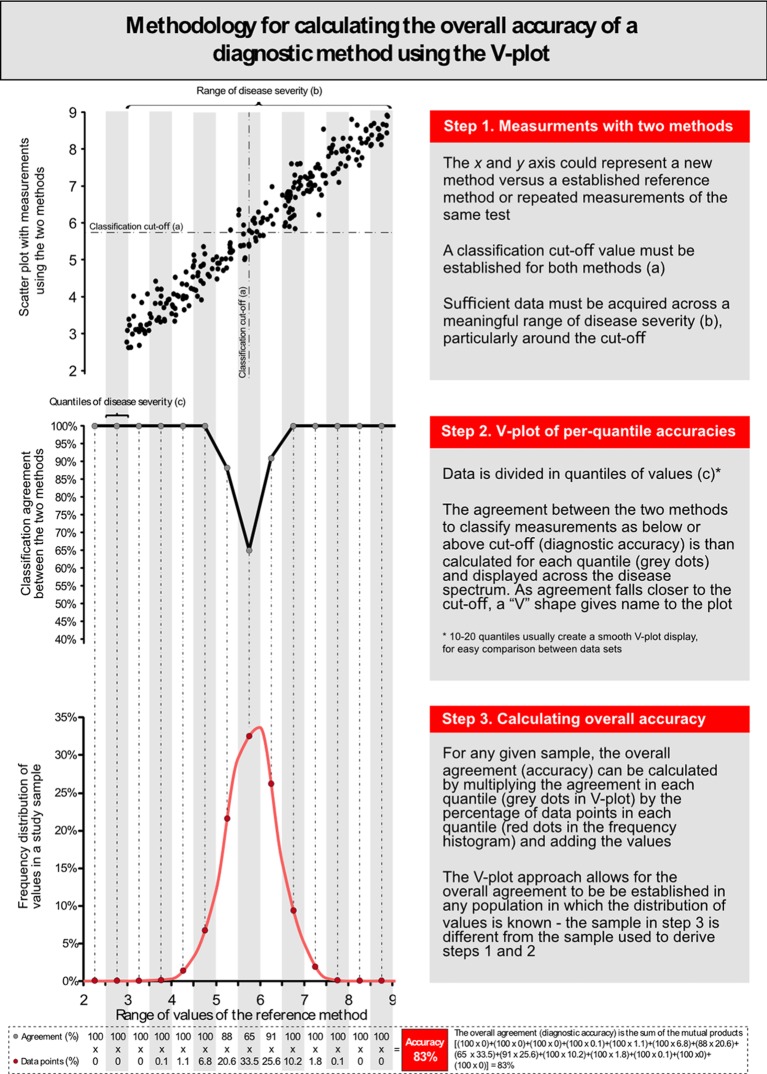
Methodology for the calculation of per-range agreement and V-plot display.

The V-plot is, therefore, a universal fingerprint of per-quantile classification agreement between two methods of measurement, which can be expressed independently of the distribution of values of the underlying sample. This can be demonstrated by displaying the V-plot from the two Chol_rapid_ studies (figure 5). Despite marked differences in the distribution of cholesterol values and very different diagnostic accuracies, the V-plots from the two studies are almost identical. This can be interpreted as the two studies showing the same degree of classification agreement between Chol_rapid_ and Chol_gold_ across the spectrum of cholesterol values. Figure 6 explains in details the steps for the display of the V-plot and for the calculation of the overall accuracy in a sample.

### Using the V-plot to derive overall accuracy in independent samples

**Figure 7:**
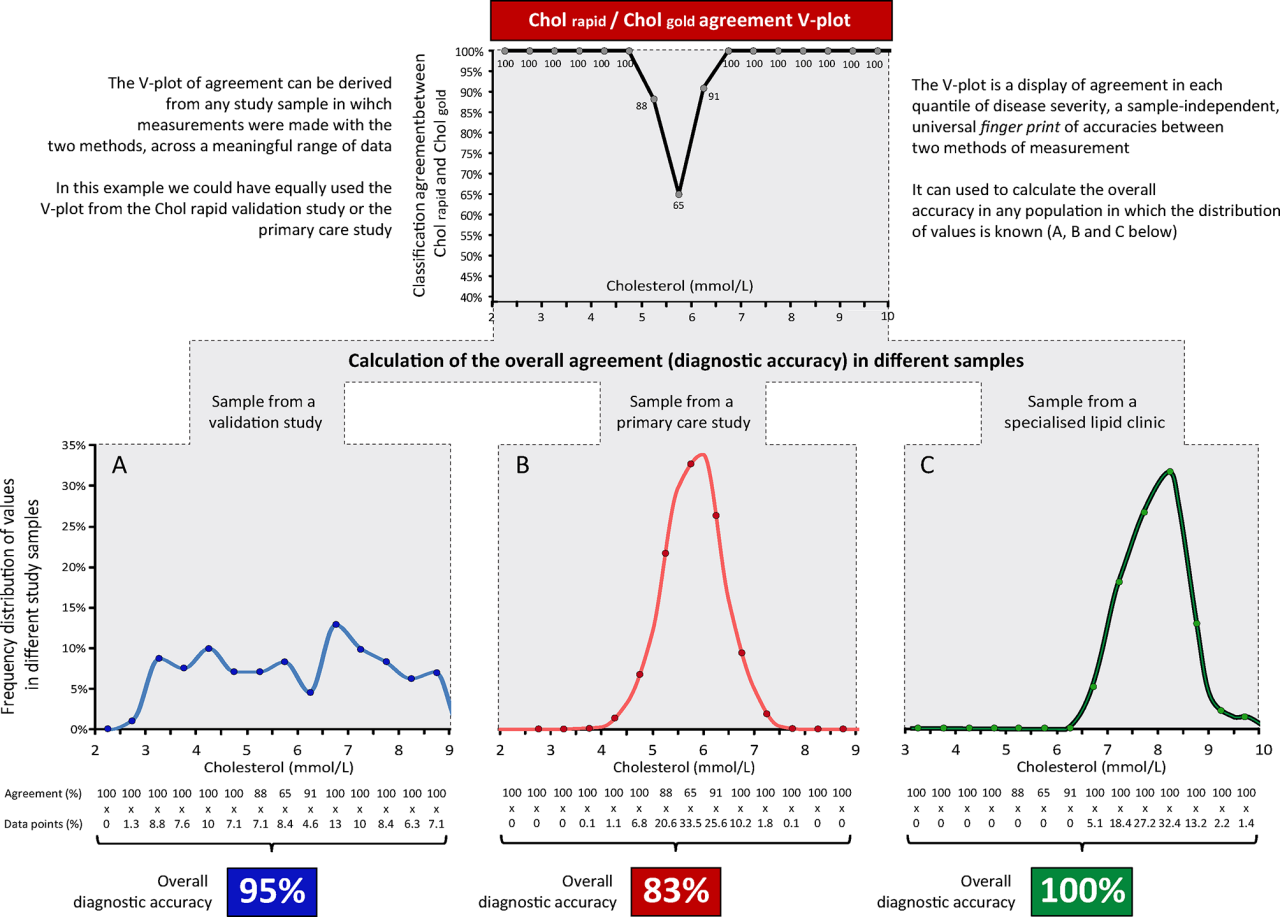
Calculating the overall accuracy in different samples using the V-plot. The V-plot agreement between Chol_rapid_ and Chol_gold_ can be derived from any study that compared the two methods (top panel). It can be used as a fingerprint of classification agreement to calculate the overall agreement between Chol_rapid_ and Chol_gold_ in any sample in which the distribution of cholesterol values is known (samples A, B and C).

Once the V-plot has been established for the relationship between any two indices, the *overall agreement* between them can be projected to any other distribution of severity. For example, once a V-plot is derived from either of the two Chol_rapid_ studies, it is possible to estimate the classification agreement between Chol_rapid_ and Chol_gold_ for a specialised outpatient lipid clinic, which is mainly formed by very high cholesterol levels (figure 7).

The simple mathematical approach to the application of the V-plot is described in figure 6. We have also made a spread sheet available, which can derive the V-plot agreement between any two methods of measurement and calculate the overall accuracy in samples with different distributions of values (online supplementary appendix).

10.1136/openhrt-2017-000663.supp1 Supplementary file 1

## Discussion

### Choosing tests based on their reported accuracy

Classification agreement between two methods of measurement is called diagnostic accuracy if one test is considered the reference gold standard. The concept of accuracy is appealing, because it gives clinicians a standardised, dimensionless measure of how good a test is (the ideal test being 100% accurate).^7^ Neither measures of the vertical scatter in a correlation plot (the SE of the estimate, a measure of numerical disagreement) nor the calculation of limits of agreement using Bland-Altman plots is as instantly appreciated by all clinicians. For instance, busy clinicians would much more confidently adopt a test that is described as 95% accurate to detect hypercholesterolaemia than choose it based on a reported 0.35 mmol/L SE of the estimate against a reference method. However, our study demonstrates the limitations of adopting tests based on published high accuracy values, without knowing the sample from which accuracy was calculated.

### Disease distribution, disease prevalence and diagnostic accuracy

The impact of disease prevalence on diagnostic accuracy and derived metrics has been previously explored.^4 8^ It is widely perceived that diagnostic metrics more closely reflect a test’s performance when disease prevalence is near 50%.^4^ Our hypothetical yet precisely controlled model of the relationship between two diagnostic methods brings interesting insights into this established statistical concept. First, our example confirms that *accuracy*, *sensitivity*, *specificity* and *likelihood ratios* are largely sample-dependent measures of a test performance. The values of all metrics changed largely between our two hypothetical samples, with less discriminative values found in the normally distributed sample formed by intermediate values of cholesterol and disease prevalence of 50% (figure 2). Second, our results highlight that the precise distribution of values across the disease spectrum (and not only the overall prevalence of disease) is what predominantly affects values of accuracy and related metrics. For instance, a sample formed by very severely diseased and very healthy individuals, away from the central cut-off (in a ‘case-control’ fashion), is likely to derive very high values of accuracy (close to 100%) with a disease prevalence that can be close to 50%.^9 10^ In contrast, samples formed by intermediate values of disease, near the boundary between normal and abnormal, can also demonstrate 50% disease prevalence yet much lower values of accuracy (figure 1). In practice, mixtures of patients from these types, and other types in between, can generate any degree of diagnostic accuracy from 100% down to approximately 50% for all diagnostic tests and definitions of severity.

### Disease severity distribution varies widely between studies

Studies that first evaluate diagnostic methods are often performed in samples whose distribution is very different from the populations in which the test will be applied in clinical practice.^11 12^ Commonly, pioneering research is performed in patients who either definitely have or definitely do not have a condition, in a case–control fashion or using a much wider spread of patients than is found in routine clinical practice.^13^ Values of accuracy and related parameters of a newly proposed methodology are universally presented, most commonly without a precise description of sample distribution of values. Rarely a frequency histogram is presented. While the desire to examine the whole spectrum is understandable, researchers may unknowingly be presenting values of a test accuracy that cannot be extrapolated to other studies nor are applicable to routine clinical practice. Physicians should realise that clinical populations often have substantially more patients in the middle zone, which inevitably lowers the accuracy of diagnostic methods.^14^ Therefore, for the relationship between any two methods of clinical measurement, there are no universal values of *diagnostic accuracy*, *sensitivity*, *specificity*, *predictive* values, *ROC curves* or even *likelihood ratios*. These parameters are only meaningful to demonstrate the *effects* of the raw measurement disagreement between the two methods (vertical scatter; figure 3) in a *specific* sample when a *specific* classification cut-off is used to define what is normal/abnormal. Although the scientific community emphasises the importance of studying new diagnostic methods in clinically relevant samples,^2 5^ this matter is rarely debated when the accuracy of new diagnostic methods are published.^15–17^

### Potential benefits of the V-plot of accuracies as a sample-independent display of the relationship between methods

In this study, we introduced the V-plot of accuracies, a simple visual approach that could help researchers and clinicians to better understand the relationship between diagnostic tests. First, the V-plot of accuracies allows interstudy comparisons, even if sample distributions differ (figure 5). For instance, if a new diagnostic method to measure cholesterol is developed, its V-plot of per-range accuracy against Chol_gold_ can be immediately derived and compared with the previously published V-plot of Chol_rapid_. While the values of overall accuracy may not be directly compared if samples are different, using the V-plot one can immediately appreciate at which point within the spectrum of cholesterol values the diagnostic accuracy of the new cholesterol test falls below a certain standard. For instance, in our two studies, Chol_rapid_ accuracy fell below 90% when cholesterol values were between 5.2 mmol/L and 6.3 mmol/L (figure 5). In practice, therefore, outside this window, Chol_rapid_ could be seen as >90% reliable and used for clinical decision making, while Chol_gold_ might still be requested in the 5.2–6.3 mmol/L uncertainty zone to confirm the diagnosis. Such hybrid approach to clinical diagnosis (trusting screening tests when results are very normal or very abnormal but requesting the gold standard in the middle zone) is often used in clinical practice instinctively.^18^ Our proposed V-plot methodology permits a formal statistical representation of such staged approach by establishing ranges of values outside which tests can be trusted to match a gold standard.

Also, as presented in figure 7, once one V-plot between methods has been established, the overall accuracy of the new test can be derived in any given sample, providing the frequency distribution of values is known. This method to derive overall accuracy in independent samples from a previous established V-plot is potentially useful if researchers or clinicians want to estimate the overall performance of new modalities without the need to collect new comparative data.

### Online appendix for V-plot derivation

We have created an online appendix in Excel format to allow readers to apply the V-plot methodology to their own datasets. Readers can adapt the calculation steps to their needs and add CIs to each quantile accuracy.

### Alternative derivations and future work on methodology

The V-plot of accuracies could be displayed in two alternative ways. First, the average between methods could be used to define disease range (the x-axis on the V-plot), if they are both felt to measure exactly the same quantity, in line with Bland-Altman plots. In our example, average values between Chol_rapid_ and Chol_gold_ could be used instead of Chol_gold_ only. The limitation of such approach is that frequency distribution of values are normally presented using histograms of the establish method and not the average between the two tests, which would limit the applicability of the V-plot to other samples. Second, the new method could be used in x-axis to define the range and quantiles (Chol_rapid_ in our example). This would allow a more directly applicable display of results as, in practice, clinicians are first faced with the results of the new test (before requesting the reference method if felt necessary). In our example, if the per-range accuracy of Chol_rapid_ was displayed using the Chol_rapid_ range of values in the x-axis, one would immediately appreciate at which point close to its cut-off it starts losing accuracy against Chol_gold_. This approach would also permit interstudy comparisons between different methods, providing the gold standard methodology was the same.

We specifically did not display CIs for each individual quantile accuracy. First, the aim of the V-plot was not to detect with precision each quantile accuracy, but instead to use the trend between quantiles to establish an overall pattern of accuracy loss from the extremes towards the intermediate range close to the cut-off. Second, the trend from the V-plot line between neighbouring quantiles act as external validity for each quantile accuracy, beyond the small sample of each quantile. As a result, to avoid overload of information to readers, we opted not to display CIs for each quantile when we first applied the methodology in real datasets.^14^ We have published an alternative V-plot methodology using logistical regression that derive 95% CIs using bootstrap.^19^ However, logistical regression is a modelling approach with intrinsic limitations, while the methodology presented in this manuscript is derived from accuracy values directly calculated in each quantile. Researchers can easily add CIs for each quantile in the spreadsheet provided (online supplementary appendix), simply by treating each quantile accuracy as a proportion and applying simple statistics.

### Limitations

The V-plot is intended to display accuracy values of a new method of measurement when quantitative variables are categorically transformed into normal and abnormal according to a pre-established cut-off. It is therefore essential for least one of the methods to be numerical so that its range of values can be displayed in the x-axis.

The V-plot describes the accuracy of a new diagnostic test to categorically match a reference method. Therefore, it assumes the ‘gold standard’ test to be an appropriate and reliable representation of the underlying variable being measured (in our example serum cholesterol).

Also, although the present hypothetical study used an example in which the numerical relationship between methods was constant across the disease spectrum (homoscedasticity assumption), the V-plot methodology could also be applied to data when heteroscedasticity is found.

How to deal with quantiles with no data points? If a quantile has no data *at the extremes* of the distribution, investigators could simply (1) not display the V-plot values at that range or (2) assume in a reasonably normally distributed sample without major heteroscedasticity that the accuracy in that range will remain 100% if it is already 100% in the immediately neighbour inner range (such as for cholesterol values below 3 in our figure 6). If a quantile has no data points *in the middle* of the disease distribution, then investigators could (1) increase their sample size, (2) decrease the number of quantiles being used, so that the absent quantile becomes merged with neighbour quantiles or (3) visually interpolate the V-plot line between the neighbouring quantiles, assuming accuracy in the absent quantile will be roughly the average of its neighbours.

This study is based on a hypothetical research scenario, and the data were created using statistical software. While this approach has limitations as data were not actually collected from patients, it allowed for a precise isolation of statistical parameters of interest and permitted a very focused analysis of the effects of data distribution on accuracy values. The V-plot methodology has already been applied to real data previously.^14 19^

The present study does not aim to discuss the clinical merits of cholesterol treatment neither the appropriateness of using a fixed cut-off for clinical decision making. The hypothetical clinical scenario of a new test to measure cholesterol was chosen to illustrate a practical application of our methodology to a wide clinical readership.

## Conclusions

For any given clinical test being compared with a gold standard, there is no universal value of diagnostic accuracy, sensitivity, specificity, predictive values, likelihood ratios or ROC curves. Accuracy will always vary progressively from almost 100% at the extremes (of health and disease) to approximately 50% (close to pure chance) near the diagnostic cut-point. Disease prevalence and the precise distribution of values in the underlying sample (extremes versus intermediate) can therefore completely control the obtained value for a test’s diagnostic accuracy. A test should not be chosen by clinicians based on a reported high accuracy value, unless the disease distribution of the study sample is known to be clinically relevant.

The V-plot of accuracies presented here exposes the variation of diagnostic accuracy along the spectrum of disease and is therefore a truly sample-independent display of categorical agreement between two methods of clinical measurement. Once derived for the relationship between two methods of measurement, the V-plot allows for the overall diagnostic accuracy to be estimated in separate samples where frequency distribution is known.

## References

[1] Leeflang MM, Deeks JJ, Gatsonis C, et al Systematic reviews of diagnostic test accuracy. Ann Intern Med 2008;149:889–97. 10.7326/0003-4819-149-12-200812160-00008 19075208 PMC2956514

[2] Mallett S, Halligan S, Thompson M, et al Interpreting diagnostic accuracy studies for patient care. BMJ 2012;345:e3999 10.1136/bmj.e3999 22750423

[3] Alberg AJ, Park JW, Hager BW, et al The use of "overall accuracy" to evaluate the validity of screening or diagnostic tests. J Gen Intern Med 2004;19(5 Pt 1):460–5. 10.1111/j.1525-1497.2004.30091.x 15109345 PMC1492250

[4] Brenner H, Gefeller O, sensitivity Vof specificity, likelihood ratios and predictive values with disease prevalence. Stat Med 1997;16:981–91. 10.1002/(SICI)1097-0258(19970515)16:9<981::AID-SIM510>3.0.CO;2-N 9160493

[5] Bossuyt PM, Reitsma JB, Bruns DE, et al STARD 2015: an updated list of essential items for reporting diagnostic accuracy studies. BMJ 2015;351:h5527 10.1136/bmj.h5527 26511519 PMC4623764

[6] Bland JM, Altman DG Statistical methods for assessing agreement between two methods of clinical measurement. Lancet 1986;1:307–10. 2868172

[7] Bossuyt PM, Reitsma JB, Bruns DE, et al Toward complete and accurate reporting of studies of diagnostic accuracy. The STARD initiative. Am J Clin Pathol 2003;119:18–22. 10.1309/8EXCCM6YR1THUBAF 12520693

[8] Willis BH Empirical evidence that disease prevalence may affect the performance of diagnostic tests with an implicit threshold: a cross-sectional study. BMJ Open 2012;2:e000746 10.1136/bmjopen-2011-000746 PMC3274715 22307105

[9] Marcos MA, Martínez E, Almela M, et al New rapid antigen test for diagnosis of pneumococcal meningitis. Lancet 2001;357:1499–500. 10.1016/S0140-6736(00)04658-4 11377604

[10] Asimaki A, Tandri H, Huang H, et al A new diagnostic test for arrhythmogenic right ventricular cardiomyopathy. N Engl J Med 2009;360:1075–84. 10.1056/NEJMoa0808138 19279339

[11] Usui S, Hara Y, Hosaki S, et al A new on-line dual enzymatic method for simultaneous quantification of cholesterol and triglycerides in lipoproteins by HPLC. J Lipid Res 2002;43:805–14. 11971952

[12] Jousilahti P, Vartiainen E, Pekkanen J, et al Serum cholesterol distribution and coronary heart disease risk: observations and predictions among middle-aged population in eastern Finland. Circulation 1998;97:1087–94. 10.1161/01.CIR.97.11.1087 9531256

[13] Pijls NH, De Bruyne B, Peels K, et al Measurement of fractional flow reserve to assess the functional severity of coronary-artery stenoses. N Engl J Med 1996;334:1703–8. 10.1056/NEJM199606273342604 8637515

[14] Petraco R, Escaned J, Sen S, et al Classification performance of instantaneous wave-free ratio (iFR) and fractional flow reserve in a clinical population of intermediate coronary stenoses: results of the ADVISE registry. EuroIntervention 2013;9:91–101. 10.4244/EIJV9I1A14 22917666

[15] Dao Q, Krishnaswamy P, Kazanegra R, et al Utility of B-type natriuretic peptide in the diagnosis of congestive heart failure in an urgent-care setting. J Am Coll Cardiol 2001;37:379–85. 10.1016/S0735-1097(00)01156-6 11216950

[16] McCullough PA, Nowak RM, McCord J, et al B-type natriuretic peptide and clinical judgment in emergency diagnosis of heart failure: analysis from Breathing Not Properly (BNP) Multinational Study. Circulation 2002;106:416–22. 10.1161/01.CIR.0000025242.79963.4C 12135939

[17] de Graaf FR, Schuijf JD, van Velzen JE, et al Diagnostic accuracy of 320-row multidetector computed tomography coronary angiography in the non-invasive evaluation of significant coronary artery disease. Eur Heart J 2010;31:1908–15. 10.1093/eurheartj/ehp571 20047991

[18] Petraco R, Park JJ, Sen S, et al Hybrid iFR-FFR decision-making strategy: implications for enhancing universal adoption of physiology-guided coronary revascularisation. EuroIntervention 2013;8:1157–65. 10.4244/EIJV8I10A179 23256988

[19] Cook CM, Petraco R, Shun-Shin MJ, et al Diagnostic Accuracy of Computed Tomography-Derived Fractional Flow Reserve : A Systematic Review. JAMA Cardiol 2017;2:803–10. 10.1001/jamacardio.2017.1314 28538960

